# Secondary abdominal pregnancy and its associated diagnostic and operative dilemma: three case reports

**DOI:** 10.4076/1752-1947-3-7382

**Published:** 2009-08-07

**Authors:** Pratiksha Gupta, Alka Sehgal, Anju Huria, Reeti Mehra

**Affiliations:** 1 Department of Obstetrics and Gynecology, Government Medical College and Hospital Sector 32B, Chandigarh, 160030, India

## Abstract

**Introduction:**

Abdominal pregnancy is extremely rare and has historically been defined as an implantation in the peritoneal cavity, exclusive of tubal, ovarian or intraligamentary pregnancy.

**Case presentations:**

Three cases are reported. All came from a lower middle-income group and all of them were subjected to surgery. The first patient was a 30-year-old woman, who was pregnant for the fourth time, who presented at 16 weeks with an abdominal pregnancy. She was admitted with constant abdominal pain and retention of urine. She was hemodynamically stable and was administered a pre-operative intramuscular injection of methotrexate. During laparotomy she had only minor blood loss, the major part of the placenta was removed easily and she did not require any blood transfusion. Serum beta human chorionic gonadotrophin values and ultrasound follow-up revealed a normal study four weeks after surgery. The second patient was a 26-year-old woman, pregnant for the third time, admitted at 14 weeks with an abdominal pregnancy with hemoperitoneum, and the third patient was a 24-year-old woman, pregnant for the first time, who presented at 36 weeks gestation. She was only diagnosed as having an abdominal pregnancy during surgery, experienced excessive blood loss and required a longer hospital stay.

**Conclusions:**

We hypothesize that treatment with pre-operative systemic methotrexate with subsequent laparotomy for removal of the fetus and placenta may minimize potential blood loss, and would be a reasonable approach in the care of a patient with an abdominal pregnancy with placental implantation to the abdominal viscera and blood vessels. This treatment option should be considered in the management of this potentially life-threatening condition. During surgery, if the placenta is attached to vital organs it should be left behind. Early diagnosis can help in reducing associated maternal morbidity and mortality.

## Introduction

Abdominal pregnancy has historically been defined as an implantation in the peritoneal cavity, exclusive of tubal, ovarian or intraligamentary pregnancy [1]. Abdominal pregnancy is a rare obstetric complication with high maternal mortality and even higher perinatal mortality, and it can be primary or secondary with the latter being the most common type. Primary peritoneal implantation is rare. Studdiford established three criteria for diagnosing primary peritoneal pregnancies: (1) normal bilateral fallopian tubes and ovaries; (2) the absence of uteroperitoneal fistula, and (3) a pregnancy related exclusively to the peritoneal surface and early enough to eliminate the possibility of secondary implantation following a primary nidation in the tube [2]. Secondary abdominal pregnancy is a condition where the embryo or fetus continues to grow in the abdominal cavity after its expulsion from the fallopian tube or other seat of its primary development. Secondary abdominal pregnancy almost always follows early rupture of a tubal ectopic pregnancy into the peritoneal cavity with the incidence being 1 in 10,000 live births [3]. Advanced abdominal pregnancy is rare and accounts for 1 in 25,000 pregnancies [4]. Risk factors for abdominal pregnancy are the same as for ectopic pregnancy and, when it is recognized, immediate laparotomy with removal of the fetus is usually recommended. As it is a life-threatening condition, expectant management carries a risk of sudden life-threatening intra-abdominal bleeding and a generally poor fetal prognosis [5]. Proper pre-operative evaluation and diagnostic techniques can help ensure a timely diagnosis, and pre-operative treatment such as embolization and methotrexate administration to minimize blood loss during surgery can facilitate maximal placental removal. Pre-operative methotrexate treatment has been described for abdominal pregnancy by Worley et al. [1] but their experience was limited to one patient who was given daily methotrexate therapy. However, sudden placental separation with acute hemorrhage developed after 4 days and they had to resort to emergency laparotomy. However, in one of our patients, pre-operative methotrexate permitted near total removal of the placenta with no major complications. Postoperative methotrexate administration has been recommended by some if the placenta is left *in situ*. Others have condemned such a practice, suggesting that rapid placental destruction leads to an accumulation of necrotic debris, inviting bacterial growth [6,7]. Rahaman et al. [6] described five patients treated postoperatively with methotrexate. Although they had rapidly declining urinary gonadotrophin levels, all five developed severe intra-abdominal infections with mortality in two patients. Methotrexate as a folate antagonist causes acute intracellular deficiency of these folate co-enzymes, thus affecting synthesis of DNA especially in rapidly multiplying cells. Methotrexate acts on rapidly dividing cells and it is likely to have limited effects on the mature placenta with its limited proliferative activity. With or without its utilization, the retained placenta will frequently undergo suppuration and require surgical removal. In cases where placental implantation has occurred in vascular areas such as the mesentery and vital organs, it has been recommended that the placenta should be left *in situ*, because surgical excision can result in uncontrollable and life-threatening hemorrhage [8]. We report a patient with a live 16-week pregnancy where a pre-operative intramuscular dose of methotrexate was given. She suffered minimal blood loss at laparotomy and major placental tissue could be removed and she did not require blood transfusion. The second patient presented as an acute emergency and surgery was undertaken immediately. In the third case the patient had repeated admissions to our hospital starting from 32 weeks onwards but the diagnosis was missed, resulting in fetal death and massive blood loss during surgery with associated morbidity.

## Case presentations

### Case 1

A 30-year-old woman (fourth gravida, G4 P2 L2 A1) presented to our hospital emergency department with amenorrhea, retention of urine, constipation and abdominal pain. On examination, mild pallor was present and she was hemodynamically stable. Abdominal examination revealed a suprapubic mass with tenderness and guarding. On vaginal examination the cervix was pushed anteriorly, the uterus was acutely retroverted, and a separate tender mass of a 16-week size was felt. Transvaginal sonogram revealed an empty uterine cavity with a live fetus of 16 weeks gestation, which was lying in the pouch of Douglas. The placenta was seen anteriorly and above the uterine fundus and no myometrium was defined around the fetus and placenta. A diagnosis of secondary abdominal pregnancy was made. The patient was given a systemic injection of methotrexate administered intramuscularly at a dose of 50 mg/m^2^. Elective laparotomy was undertaken 48 hours later. During surgery the fetus and gestational sac were seen lying in the pouch of Douglas and the placenta was adherent to the right fallopian tube, the fundus of the uterus, the omentum and the bowel. Extraction of the fetus with placental removal and partial omentectomy was carried out. One-third of the placenta was left behind as it was judged not safe to remove it. Blood loss during surgery was around 200 mL and the patient required no transfusion. The postoperative period was uneventful and she was discharged on the 10th postoperative day. Serum beta human chorionic gonadotrophin levels returned to normal after three weeks and ultrasound examination revealed complete resorption of the placenta at around four weeks.

### Case 2

A 26-year-old woman, third gravida, with two previous full-term vaginal deliveries, was admitted on 13th May 2008. Her last menstrual period was on 26th February 2008 and bilateral tubal ligation was done in March 2008. Her symptomatology started a month after ligation when she had lower abdominal pain. Her urine pregnancy test was positive on 28th April and dilatation and evacuation was performed on 29th April. Two days before admission to hospital her pain increased in severity and was associated with fever and painful micturition. At admission she had moderate anemia, tachycardia and hypotension. Abdominal examination revealed free fluid in the abdominal cavity. Per-vaginal examination revealed a normal size uterus with tenderness and an ill-defined mass in the right adnexa. An ultrasound showed an intraperitoneal fluid collection, a normal size uterus and a live fetus of 14-weeks' gestation, which was lying in the pouch of Douglas. Abdominal paracentesis confirmed hemoperitoneum. Intra-operative findings revealed the placenta to be attached to the omentum, the bowel and the posterior surface and the right cornua of the uterus. Partial omentectomy was performed and part of the placenta was left behind. Two units of blood were transfused. The patient did well postoperatively and complete placental resorption took approximately six weeks.

### Case 3

A 24-year-old woman, primigravida, was admitted to our hospital with abdominal pain at 32 weeks of pregnancy with anemia and mild abdominal pain. She had two units of blood for her anemia and ultrasound examination reported a live intrauterine pregnancy with placenta praevia. She was discharged after her pain subsided. This patient had repeated hospital admissions starting at around 14 weeks of gestational age for constant abdominal pain, for which uterine colic was diagnosed and analgesics were prescribed each time. She had sudden loss of fetal movement at 39 weeks of pregnancy and ultrasound revealed intrauterine fetal demise with a low-lying placenta. Spontaneous onset of labor did not happen, and at 41+1 weeks of pregnancy induction of labor was started. The cervix failed to ripen in spite of maximum doses of prostaglandin and her uterus also failed to stimulate with oxytocin. She was then taken to surgery for caesarian section for failed induction. During the surgery secondary abdominal pregnancy was diagnosed with the fetus seen lying in the abdominal cavity and the placenta was adherent to the omentum and the bowel. The fetus was extracted and during removal of the placenta the patient had torrential bleeding so that only one-quarter of the placenta could be removed. The bleeding was controlled by packing and pressure. The patient received seven units of whole blood during the surgery. She was discharged on postoperative day 20 without any major complications. Follow-up was done by measuring serum beta human chorionic gonadotrophin levels and ultrasound examination; it took six months for complete absorption of the placenta.

## Discussion

Abdominal pregnancy is a rare obstetric complication with high maternal and perinatal mortality. Ultrasound, magnetic resonance imaging (MRI), computed tomography (CT) scan and laparotomy can help in differentiating between primary and secondary abdominal pregnancy [9]. As early rupture of tubal ectopic pregnancy is the usual antecedent of a secondary abdominal pregnancy, a suggestive history can usually be obtained. These include spotting or irregular bleeding along with abdominal pain, nausea, vomiting, flatulence, constipation, diarrhea and abdominal pain, all in varying degrees. Fetal malpresentation, extreme anterior displacement of the cervix, failure of spontaneous onset of labor and artificial induction of labor are common complications. Appreciable cervical effacement is also unusual in these patients. Small fetal parts may be palpated through the vaginal fornices and identified clearly outside the uterus [5]. The patient with an abdominal pregnancy typically presents with constant abdominal pain; two of our patients sought medical attention for this and initial evaluation showed them to be hemodynamically stable. About 50% of diagnoses are missed on ultrasound [10] but MRI and CT are both excellent diagnostic tools to diagnose secondary abdominal pregnancy [3,5]. In our first and second patients, the clinical diagnoses were supported by ultrasound examination, but the third patient was misdiagnosed, ending in fetal death. She had typical signs and symptoms of constant abdominal pain, progressive anemia and sudden fetal death. She also had failed induction of labor, which could have clinched the diagnosis but it was missed. CT scan or MRI could have diagnosed this patient pre-operatively and pre-operative methotrexate could have been planned and resulted in less blood loss during placental separation and hence reduced maternal morbidity. It should be noted that even in cases where it is the surgeon's intent to leave the placenta *in situ*, serious hemorrhage can occur because of inadvertent disruption of the placental blood supply in the process of fetal removal. This is likely due to the unpredictable nature of the placental blood supply, as well as the difficult surgical challenges presented by extensive adhesion formation typically found in abdominal pregnancy. Successful control of hemorrhage after removal of the fetus and placenta at laparotomy depends upon the various treatment protocols used pre- and intra-operatively. Even pre-operative embolization of vessels supplying the placental bed has been tried by some surgeons, thus decreasing intra-operative blood loss associated with removal of the fetus and placenta [6].

During laparotomy the clinician must make a decision concerning the fate of the placenta. Postoperative maternal morbidity will probably be lessened by total removal of the placenta if this is technically feasible and this should be possible using proper pre-operative treatment modalities such as embolization or systemic methotrexate. If vascular attachment involves major vessels or vital structures, the organ should be left undisturbed [8]. Postoperative methotrexate has been administered by some for placental absorption but it leads to accumulation of necrotic tissue due to accelerated placental absorption and increases morbidity [5-7]. Retention of the placenta *in situ* is not without its attendant risks and postoperative morbidity can be substantial. Secondary hemorrhage, abscess formation, paralytic ileus, bowel obstruction, pre-eclampsia and eclampsia have all been reported as complications of leaving the placenta *in situ*[11]. Any modality which can offer a reduction of intra-operative vascularity and hence blood loss would be a boon for these patients. Where the facility for embolization is not easily accessible, injectable methotrexate is an easy, cheap and relatively safer option. Resorption of a placenta left *in situ* has been reported to be a slow process and can be followed either by sonography, MRI or CT scan. Visualization of a hyperechoic placental mass has been reported as late as five years after delivery [12]. Although no consensus regarding the treatment of the placenta in abdominal pregnancy has been established, most authors advocate leaving the placenta *in situ* unless the surgeon can be confidently assured that the entire blood supply to the placental bed can be surgically ligated without loss of excessive amounts of blood, and the need for extensive blood replacement therapy. Pre-operative arterial embolization has also been advocated by some [6]. We successfully managed to remove more than half of a placenta without significant blood loss and without the patient requiring blood transfusion. A literature review shows that in one report an 18-week abdominal pregnancy was managed by percutaneous sonographically guided intracardiac injection of potassium chloride followed by uncomplicated laparotomy with complete removal of the fetus and placenta [13]. In another study, the patient was managed at 19 weeks of pregnancy by sonographically guided intracardiac potassium chloride with an intramuscular injection of 50 mg/m^2^ methotrexate. This patient had no surgical intervention and complete resorption occurred over a period of 1.5 years [11].

Many authors have described pregnancies carried to term with live births, various fetal congenital malformations, poor fetal outcome and high neonatal mortality [4,5] and even minimally invasive surgeries have been tried up to the second trimester of pregnancy [6,14,15].

The treatment options for patients with abdominal pregnancies are dependent on various factors and the availability of resources. Pre-operative arterial embolization would have been the first choice in our patients but due to lack of facilities we resorted to pre-operative intramuscular methotrexate. Other pre-operative considerations include the insertion of ureteric catheters, bowel preparation, assurance of sufficient blood products and the availability of a multidisciplinary surgical team. Should such resources not be available, elective transfer of a woman with a known advanced extra-uterine pregnancy to a tertiary care facility is appropriate. If discovery is not made until attempted cesarean delivery, even then a safer alternative would be to defer delivery if possible, close the abdominal incision, and transfer the woman to an appropriate hospital. This could be done even after the delivery of the fetus or neonate with the placenta left *in situ* if there was no bleeding [1].

We propose that pre-operative systemic methotrexate with subsequent laparotomy for removal of the fetus and placenta may minimize potential blood loss, and may be a reasonable approach in the care of a patient with an abdominal pregnancy with placental implantation to abdominal viscera and blood vessels. This treatment option could be considered in the management of this potentially life-threatening condition, but more reports and experience are required. Proper pre-operative evaluation with appropriate diagnostic techniques can help with a timely diagnosis, and pre-operative treatment such as methotrexate administration to minimize blood loss at surgery can facilitate maximal placental removal. As the placenta continues to grow throughout the pregnancy methotrexate is recommended at all gestational ages.

## Conclusion

Proper pre-operative evaluation and pre-operative systemic methotrexate, assurance of sufficient blood products, availability of a multidisciplinary surgical team and proper operative techniques in managing abdominal pregnancy can reduce maternal morbidity.

## Abbreviations

CT: computed tomography; MRI: magnetic resonance imaging.

## Consent

Written informed consent was obtained from the patients for publication of these case reports and accompanying images. A copy of the written consents is available for review by the Editor in-Chief of this journal.

## Competing interests

The authors declare that they have no competing interests.

## Authors' contributions

PG, AS, AH and RM all contributed equally to the surgeries, interpretation of the patients' data and formation of the manuscript. PG was a major contributor in writing the manuscript. All authors read and approved the final manuscript.
